# Targeting Soluble VCAM1 and GSK3β Improves Cerebrovascular Function and Reduces Stroke Pathology in Diabetic Mice

**DOI:** 10.3390/cells15050455

**Published:** 2026-03-04

**Authors:** Masuma Akter Brishti, Mousumi Mandal, Udai Pratap Singh, Tauheed Ishrat, M. Dennis Leo

**Affiliations:** 1 Department of Pharmaceutical Sciences, College of Pharmacy, University of Tennessee Health Science Center, Memphis, TN 38163, USA; mbrisht1@uthsc.edu (M.A.B.);; 2 Department of Anatomy and Neurobiology, College of Medicine, University of Tennessee Health Science Center, Memphis, TN 38163, USA

**Keywords:** type 2 diabetes, HFD-STZ model, insulin resistance, mast cells, soluble vascular cell adhesion molecule-1, Glycogen Synthase Kinase 3 beta

## Abstract

**Highlights:**

**What are the main findings?**

- Insulin resistance and soluble VCAM1 act through complementary pathways in mast cells to elevate circulating histamine levels, leading to cerebrovascular dysfunction.
- Dual targeting of sVCAM1 and GSK3β lowered histamine, improved endothelial barrier metrics and cerebral artery tone, and reduced infarct size and edema.

**What are the implications of the main findings?**

- Treating diabetic cerebrovascular dysfunction requires pathway-specific interventions beyond glycemic lowering.
- Blocking sVCAM1 signaling and inhibiting GSK3β represent complementary therapeutic strategies to protect vascular function in the diabetic brain.

**Abstract:**

Type 2 diabetes (T2D) features insulin resistance that promotes cerebrovascular injury, yet the immune signals linking metabolic stress to vascular dysfunction remain unclear. We tested the hypothesis that insulin resistance and soluble vascular cell adhesion molecule-1 (sVCAM1) act through complementary pathways in mast cells (MCs) to raise circulating histamine levels and impair cerebral vascular function. In a high-fat diet (HFD) plus low-dose streptozotocin (STZ) model, plasma histamine rose sharply after the onset of insulin resistance and remained elevated. Plasma sVCAM1 levels also increased after insulin resistance. In vitro, recombinant sVCAM1 upregulated histidine decarboxylase (HDC) in native MCs in a dose-dependent manner, indicating a shift toward histamine synthesis, but did not enhance degranulation. In contrast, pharmacological inhibition of Akt with MK2206 activated Glycogen Synthase Kinase 3 beta (GSK3β) and increased MC degranulation without affecting HDC expression. Diabetic endothelial cell monolayers exhibited a ~twofold reduction in transendothelial electrical resistance consistent with impaired blood–brain barrier (BBB) integrity. Diabetic cerebral arteries showed receptor remodeling that favored constriction with histamine H1 receptor (H1R) expression increasing in vascular smooth muscle, while endothelial H1R and histamine H2 receptor (H2R) decreased. Functionally, insulin treatment lowered HOMA2-IR in T2D mice but did not restore cerebral artery myogenic tone or improve stroke outcomes after distal middle cerebral artery occlusion (dMCAO). Neutralizing VCAM1 with a monoclonal antibody reduced circulating sVCAM1 and histamine levels, and, together with the GSK3β inhibitor Tideglusib, stabilized MCs, normalized cerebral artery tone, and reduced post-MCAO infarct size and edema. These findings identify two distinct yet complementary mast cell pathways in T2D, highlight an immune-vascular interface that drives cerebrovascular dysfunction, and propose sVCAM1 blockade plus GSK3β inhibition as rational strategies to protect cerebral vascular function in the diabetic brain.

## 1. Introduction

Type 2 diabetes (T2D) is a rapidly escalating global health concern, currently affecting more than 500 million people worldwide [1]. A core feature of T2D is insulin resistance, which not only impairs glucose metabolism but also contributes to widespread vascular complications [1,2]. Among these, diabetic vasculopathy (DVD), marked by endothelial dysfunction, impaired vascular reactivity, and chronic low-grade inflammation, is a major driver of morbidity and mortality [3,4]. Despite advances in glycemic control, the risk of stroke and cardiovascular disease remains disproportionately high [5] in individuals with T2D, highlighting the need for new insights into disease mechanisms.

While classical contributors to vascular injury in diabetes include oxidative stress, nitric oxide deficiency, and leukocyte adhesion, recent evidence suggests that mast cells (MCs) could play a role in propagating vascular inflammation [6,7,8]. MCs are tissue-resident immune cells capable of rapid degranulation and release of pro-inflammatory mediators [9,10,11]. Their activation has been implicated in endothelial disruption, vascular permeability, and thrombosis [9,12,13]. Although MCs are increasingly recognized as players in metabolic inflammation, their role in the context of T2D-induced cerebral vascular dysfunction remains poorly defined. Histamine, a major mediator stored in and released by MCs, plays a significant role in regulating vascular tone, increasing permeability, and promoting leukocyte recruitment [14,15]. Elevated histamine levels have been observed in diabetic conditions and may contribute to the amplification of vascular injury [16,17,18]. However, the upstream signals driving histamine synthesis and MC activation in T2D, particularly in the cerebrovascular context, remain incompletely understood.

Soluble vascular cell adhesion molecule-1 (sVCAM1) is a circulating form of VCAM-1, an endothelial adhesion molecule upregulated in response to inflammatory stimuli. sVCAM-1 levels are elevated in T2D and have been associated with increased cardiovascular risk, including stroke [19,20,21,22]. While sVCAM-1 is widely used as a biomarker of endothelial activation, emerging studies suggest it may also have biological effects beyond passive circulation. MCs express integrins such as α4β1 (VLA-4), which can bind to VCAM-1 [23,24], raising the possibility that sVCAM-1 may engage these receptors and modulate MC function directly. Previously, we reported that insulin resistance (IR) induces endothelial cell VCAM1 ectodomain shedding, increasing circulating sVCAM1 levels in T2D mice [25]. Our data here show that plasma histamine rises in T2D mice after the onset of IR. Because MCs are the principal source of circulating histamine [14,15], we hypothesized that IR and sVCAM1 converge on MCs to increase histamine production and release, thereby promoting cerebrovascular dysfunction.

Our results indicate two distinct but convergent signals that elevate histamine from MCs. sVCAM1 increased histidine decarboxylase (HDC) in MCs, thereby promoting histamine synthesis, without increasing degranulation. In contrast, insulin resistance increased degranulation by activating Glycogen Synthase Kinase 3 beta (GSK3β), a kinase usually restrained by Akt-dependent Ser9 phosphorylation [26,27,28,29,30,31,32]. We have previously also shown that the onset of insulin resistance leads to dysfunctional Akt signaling [33]. Here, we found that IR leads to higher GSK3β activity and greater MC degranulation. Together, these pathways raised plasma histamine during T2D. At the arterial level, we observed receptor remodeling in diabetic cerebral arteries that favors constriction: H1R was increased in vascular smooth muscle, while endothelial H1R and H2R were reduced, aligning with elevated myogenic tone and impaired blood-brain-barrier (BBB) resistance, in addition to elevated stroke pathology following the middle cerebral artery occlusion (MCAO) method. Functionally, we observed that glycemic control alone did not correct these abnormalities. We tested a novel approach using a monoclonal VCAM1 antibody (VmAb) in combination with a GSK3β inhibitor, Tideglusib [25,34], and showed that these treatments lowered histamine levels, stabilized MCs, improved cerebral arterial tone, and reduced infarct size and edema. These findings provide novel insights into the immune-vascular interface in T2D and suggest sVCAM1 blockade and GSK3β inhibition as complementary therapeutic strategies for diabetic cerebrovascular disease.

## 2. Materials and Methods

### 2.1. Animal Use

All procedures followed protocols approved by the UTHSC Institutional Animal Care and Use Committee. Mice strains used include: C57BL/6J (WT), *Lepr^db^* (*db***/***db*), and Ins2*^Akita^* (T1D-Akita), all purchased from The Jackson Laboratory (Bar Harbor, ME, USA). A type 2 diabetes model was established using a high-fat diet (HFD) combined with low-dose streptozotocin (STZ) and has been previously validated by our group [25,33,35]. Because the high-fat diet plus streptozotocin protocol is unreliable in C57BL/6J female mice, all experiments were performed in male mice, and all other strains were matched accordingly. All mice assigned to the T2D cohort received a high-fat diet (HFD) combined with low-dose streptozotocin (STZ). At 6 weeks of age, mice were placed on HFD (TD.88137, Inotiv, Chicago, IL, USA). After 8 weeks on this diet, animals received STZ (Sigma-Aldrich, St. Louis, MO, USA) intraperitoneally at 40 mg/kg/day for 4 consecutive days and remained on HFD for an additional 2–4 weeks. Mice were humanely euthanized at defined time points, as required by the specific experiment. Blood glucose was measured with AlphaTrak2 test strips, plasma insulin was quantified using a mouse insulin ELISA kit (Crystal Chem, Elk Grove Village, IL, USA), and plasma histamine was quantified by a competitive ELISA kit (ThermoFisher Scientific Inc., Waltham, MA, USA). Insulin resistance was estimated using the HOMA2 calculator from the Diabetes Trials Unit, University of Oxford. For treatment, T2D mice were randomized one week after the final streptozotocin dose into three treatment arms: long-acting insulin (LA-I) alone, or LA-I with VmAb plus Tideglusib. LA-I used was insulin glargine U100 (Lantus, Sanofi Aventis, Bridgewater, NJ, USA) given once daily for 15 days by subcutaneous injection [36,37]. Doses were titrated every 3 to 4 days to maintain fasting glucose between 120 and 150 mg/dL and avoid hypoglycemia. VmAb was an in vivo-grade, low-endotoxin, carrier-free anti-mouse VCAM1 antibody (#BE0027, BioXCell, Lebanon, NH, USA) administered intraperitoneally as a 15 mg/kg loading dose on day 1, followed by 10 mg/kg on days 4, 8, and 12. Tideglusib was given intraperitoneally at 10 mg/kg/day for 15 days, prepared in 4% DMSO in corn oil. Vehicle controls received rat IgG (10 mg/kg) and a DMSO + corn oil mixture to match the treatment group. Injection sites were rotated. Body weight and fasting glucose were monitored at least five days per week. Middle cerebral artery occlusion, cerebral artery contractility studies, and final tissue collection were performed on day 16 after treatment initiation. Animals were included based on strain, age, sex, and baseline health; no additional inclusion criteria were applied. Exclusion criteria were established a priori and included severe lethargy or moribund state, greater than 15% weight loss, sustained hypoglycemia, inability to maintain normothermia, or filament malposition during MCAO. Data points were excluded only for predefined technical failure. After baseline measurements, mice were allocated to groups using a random sequence stratified by body weight and fasting glucose to balance baseline metabolic status. To minimize confounders, cages were distributed across shelves and racks to balance room position, light, and airflow. The order of procedures (glucose testing, myography, MCAO) was counterbalanced daily and rotated between groups. Littermates were split across groups. Group assignments were generated by one team member not involved in the experiments. Personnel performing treatments were aware of allocation for dosing accuracy, but outcome assessors were blinded for TEER, Western blots, histamine, and sVCAM1 assays, myography, and infarct quantification. Expected events occurred at low frequency and were managed per protocol, including occasional diabetes-related lethargy or weight loss and transient hypoglycemia during LA-I titration. Data analysis was conducted on coded datasets, and unblinding occurred only after statistics were finalized.

### 2.2. Peritoneal Mast Cell Isolation

All MCs used here were mouse peritoneal mast cells that were isolated as described previously [38,39]. Experimental mice were euthanized first, and then the peritoneal cavity was filled with ice-cold PBS containing 2 U/mL heparin. The resulting solution with cells was collected into 50 mL Eppendorf tubes. Samples were pooled within each experimental group, filtered through a cell strainer, and pelleted by centrifugation at 400g for 5 min. Erythrocytes were lysed, and total peritoneal cells were seeded in RPMI 1640 containing 10% FBS, 1% penicillin-streptomycin, 2 mM L-glutamine, 10 ng/mL recombinant mouse IL-3, and 30ng/mL recombinant mouse Stem Cell Factor (SCF). After 24 h, adherent cells were discarded, and nonadherent cells were maintained at 37 °C and 5% CO_2_, with medium changes every 2 to 3 days. Cultures reached greater than 90% purity by day 7 to 10, as confirmed by flow cytometry using c-Kit and FcεRIα (both from BioLegend Inc., San Diego, CA, USA).

### 2.3. β-Hexosaminidase Assay

MCs were sensitized overnight with anti-DNP IgE (clone SPE 7, MilliporeSigma, St. Louis, MO, USA) at 0.5 μg/mL in complete medium at 37 °C and 5% CO_2_. Cells were washed three times and resuspended in Tyrode’s buffer containing Ca^2+^ and Mg^2+^. For stimulation, 1 × 10^5^ cells in 100 μL were plated per well and equilibrated for 10 min at 37 °C, then challenged with DNP-HSA (MilliporeSigma, St. Louis, MO, USA) at 10 to 100 ng/mL for 30 min. Parallel wells received buffer only for spontaneous release or 0.1 percent Triton X-100 at the end of the incubation for maximum release. Plates were centrifuged at 300× *g* for 5 min. Supernatants were transferred to a fresh plate, and pellets were lysed in 0.1 percent Triton X-100 for 15 min. Beta hexosaminidase activity was quantified by adding an equal volume of 1 mM p-nitrophenyl N-acetyl-β-D-glucosaminide (pNAG, MilliporeSigma, St. Louis, MO, USA) prepared in 0.1 M citrate buffer, pH 4.5, followed by incubation at 37 °C for 45 to 60 min. Reactions were stopped with 0.2 M glycine carbonate, pH 10.4, and absorbance was read at 405 nm. Percent degranulation was calculated as 100 times supernatant activity divided by the sum of supernatant plus lysate activity, after subtraction of spontaneous release.

### 2.4. Transendothelial Electrical Resistance (TEER) Measurements

Endothelial cells (ECs) were isolated from freshly dissociated control or diabetic mouse cerebral arteries using the Endothelial Cell Isolation Kit (Miltenyi Biotec, Gaithersburg, MD, USA) as per the manufacturer’s workflow. Single-cell suspensions were prepared by enzymatic digestion of tissue and then magnetically labeled. For positive selection, labeled cells were applied to a CD31 MicroBead MS column (Miltenyi Biotec, Gaithersburg, MD, USA) placed in a magnetic rack. The column was rinsed twice with PBS containing 0.5 percent BSA to remove unlabeled cells, and CD31-positive cells were eluted after the column was removed from the magnet. Purified cells were plated in EC basal medium (PromoCell, Heidelberg, Germany) supplemented with the manufacturer’s growth supplement mix and antibiotics. Medium was replaced on day 2 and then on days 4 and 7. Cultures typically reached confluence by day 7. Primary ECs were then dissociated and plated on 0.4 μm pore size, tissue culture-treated transwell inserts (Millipore Sigma, St. Louis, MO, USA) at 105 cells per insert in endothelial growth medium. Monolayers were cultured at 37 °C, 5% CO_2_ until reaching stable confluence, as verified by phase-contrast microscopy and plateaued resistance. TEER was measured using the Millicell ERS3.0 digital Volt-Ohmmeter (MilliporeSigma, St. Louis, MO, USA). Blank inserts containing medium only were measured in parallel. TEER was calculated as (measured resistance minus blank) multiplied by membrane area and reported in ohm·cm^2^. All measurements were performed at 37 °C in triplicate and averaged within biological replicates.

### 2.5. Western Blotting

Western blotting for total protein was performed according to standard protocols. Cerebral arterial segments were pooled from 2–3 mice for experiments measuring protein abundance. Proteins were separated on 8% SDS-polyacrylamide gels and transferred onto nitrocellulose membranes. Membranes were blocked with 5% nonfat milk and incubated with one of the following primary antibodies overnight at 4 °C: anti-insulin receptor β (#3025), phospho-GSK3β (Ser9; #5558), and histamine H1 Receptor (#55550) were from Cell Signaling, Inc., anti-histidine decarboxylase (#EPR26392-79) was from Abcam Inc. (Waltham, MA, USA), histamine H2 receptor (#NB600-812) was from Novus Biologicals (Centennial, CO, USA), and anti-actin (#MAB1501) was from MilliporeSigma (St. Louis, MO, USA). Membranes were washed and incubated with horseradish peroxidase-conjugated secondary antibodies at room temperature. Blots were physically cut to allow probing of multiple proteins without stripping. All primary antibodies were used at 1:500 dilution, except anti-Actin, which was used at 1:5000. All secondary antibodies were used at 1:5000 dilution. Protein bands were imaged using a ChemiDoc gel imaging system (Biorad Inc. Hercules, CA, USA), quantified using Quantity One software v 4.6 (Biorad Inc.), and normalized to Actin.

### 2.6. Akt Kinase Activity Assay

Mast cell lysates were analyzed for total Akt kinase activity using a commercial kit (Akt Kinase Activity Assay, Abcam, ab139436) according to the manufacturer’s protocol. Briefly, cells were homogenized to generate lysates, which were then incubated with ATP for 90 min at 30 °C. The reaction mixture was transferred to the ELISA plate and incubated with the kit’s phospho-specific substrate antibody for 60 min at room temperature. An HRP-conjugated anti-rabbit IgG was applied for 30 min, followed by development with TMB for 60 min. The reaction was terminated with the supplied stop solution, and absorbance was read at 450 nm on a plate reader (BioTek, Winooski, VT, USA). Akt activity was quantified by comparison to a standard curve prepared using the kit reagents.

### 2.7. Pressurized Artery Myography

Endothelium-intact middle cerebral arteries were dissected from mouse brains and cleaned of surrounding tissue. Arterial segments (1–2 mm long) were collected from control and untreated T2D mice at 2-week intervals from week 16 to 22. For the treatment groups, arteries were collected one week into the treatment period (18 weeks) and twice (20 and 21 weeks) after the end of the treatment period. Segments were then cannulated at each end in a perfusion chamber (Living Systems Instrumentation) containing physiological saline, gassed with 21% O_2_, 5% CO_2_, and 74% N_2_ (pH 7.4), and maintained at 37 °C. Intravascular pressure was monitored with a pressure transducer and maintained at 60 mmHg. Changes in arterial diameter were measured at 1 Hz using a CCD camera attached to a Nikon TS100-F microscope and the automatic edge-detection function of IonWizard software v. 6 (Ionoptix, Milton, MA, USA). Myogenic tone was calculated as 100 × (1 − Dactive/Dpassive), where Dactive is the active arterial diameter and Dpassive is the diameter determined in the presence of Ca^2+^-free physiological saline supplemented with 5 mM EGTA.

### 2.8. Middle Cerebral Artery Occlusion (MCAO) Stroke Model

Adult mice underwent MCAO using the intraluminal suture model as described previously [40,41]. Mice were anesthetized with 2–5% isoflurane in oxygen and positioned under a stereomicroscope. The distal middle cerebral artery was identified just proximal to the bifurcation into frontal and parietal branches. For transient dMCAO, the vessel was occluded using a 9–0 nylon ligature for 45 min, followed by removal to allow reperfusion. Core temperature was maintained at 37 °C during surgery and recovery. Lidocaine 5% ointment was applied to the surgical site. Animals were returned to warmed cages and monitored for signs of distress for 24 h. Animals received buprenorphine (0.05 mg/kg, sc) before reperfusion and at 12 h after MCAO. After 24 h post-reperfusion, animals were euthanized, and brains were rapidly removed, chilled, and sectioned into 2 mm coronal slices. Slices were incubated in 2,3,5-triphenyltetrazolium chloride (TTC; MilliporeSigma, USA) to visualize infarcted tissue. Infarcted and total hemispheric areas were quantified with ImageJ v1.54r. Edema-corrected infarct volume was calculated, and hemispheric edema was expressed as the difference in hemispheric area between the ischemic and contralateral sides.

### 2.9. Statistical Analysis

Statistical analyses were performed in OriginLab v10 and GraphPad InStat v3.1. Group sizes were set by a priori power calculation for the primary endpoint (cerebral artery myogenic tone), targeting 80% power at α = 0.05 to detect a 25% difference based on pilot variance. For secondary readouts, sample sizes followed the same variance assumptions and prior work in this model to ensure adequate power while minimizing animal use. Outcome measures were as follows, for primary vascular physiology: middle cerebral artery myogenic tone by pressurized myography, for barrier integrity: TEER of primary cerebral endothelial monolayers and junctional proteins (ZO-1, claudin-5) by immunoblot, for MC biology: degranulation by β-hexosaminidase release, HDC protein by Western blot, for circulating mediators: plasma histamine and sVCAM1 by ELISA, for receptor profiling: H1R/H2R protein in endothelium and smooth muscle, and for stroke outcomes: edema-corrected infarct volume after dMCAO. Some assays used pooled samples, combining 2–3 mice to generate a single biological replicate. Across all cohorts and experiments, 452 mice were used. Data are presented as mean ± SE. Two group comparisons used a two-tailed Student’s *t*-test or a Mann–Whitney U test when distributional assumptions were not met. For comparisons involving more than two groups, one-way ANOVA was applied, followed by Bonferroni post hoc testing. A *p*-value < 0.05 was considered statistically significant.

## 3. Results

### 3.1. Validation of a T2D Mouse Model

**Figure 1:**
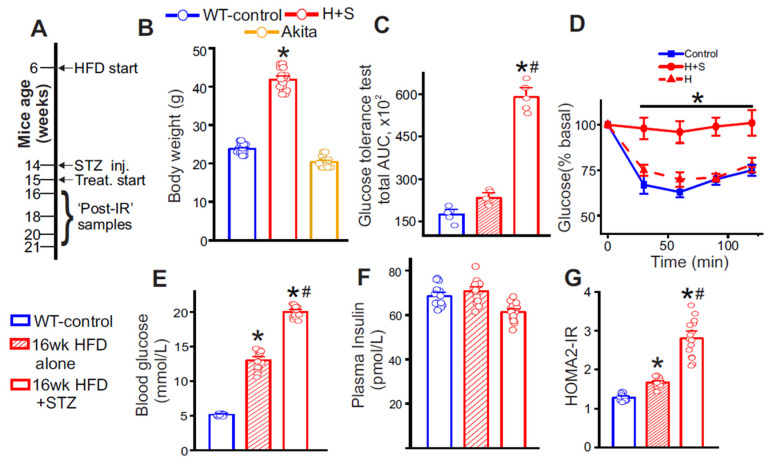
High-fat diet (HFD) + low-dose streptozotocin (STZ) induces insulin resistance in C57BL/6 mice. (**A**) Schematic of the diet/STZ protocol and treatment schedules followed. (**B**) End-point body weight (in g) from 21-week-old mice. n = 15 for each. * *p* < 0.05 vs. control. (**C**) Glucose Tolerance test (GTT) and (**D**) Insulin Tolerance Test (ITT), in control, HFD only mice (H, week 16), and after STZ injections (H + S, week 16). n = 4–5 each, * *p* < 0.05 vs. respective control, # *p* < 0.05 vs. week 16 HFD only. (**E**) fasting blood glucose (in mmol/L), (**F**) Plasma insulin (in pmol/L), and (**G**) HOMA2-IR of all mice recorded at 16 weeks of age. n = 15 each, * *p* < 0.05 vs. control and # *p* < 0.05 vs. HFD only.

Our T2D model combined a high-fat diet with low-dose streptozotocin. In T2D mice subjected to this protocol, endpoint body weight was significantly higher (Figure 1B), whereas T1D-Akita mice showed no increase (Figure 1B). Blood glucose rose after STZ compared with matched HFD-alone time points (Figure 1E). Plasma insulin levels were not significantly altered (Figure 1F), consistent with prior work in this model showing that circulating insulin levels alone are not a reliable determinant of insulin resistance [42,43]. Glucose and insulin tolerance testing were performed according to standard methodologies [44]. Results indicate that glucose tolerance worsened in HFD-fed mice after STZ administration relative to pre-STZ testing (Figure 1C). Consistent with this, the HFD + STZ group displayed reduced insulin sensitivity compared with HFD-only controls, evidenced by a smaller decrease in blood glucose during the insulin tolerance test (Figure 1D). Insulin sensitivity, assessed by HOMA2-IR, was significantly worse after the HFD + STZ protocol than with HFD alone (Figure 1G). Thus, mice developed robust insulin resistance (IR) with this inducible T2D regimen.

### 3.2. Circulating Plasma Histamine and sVCAM1 Levels Are Significantly Elevated in T2D Mice

**Figure 2:**
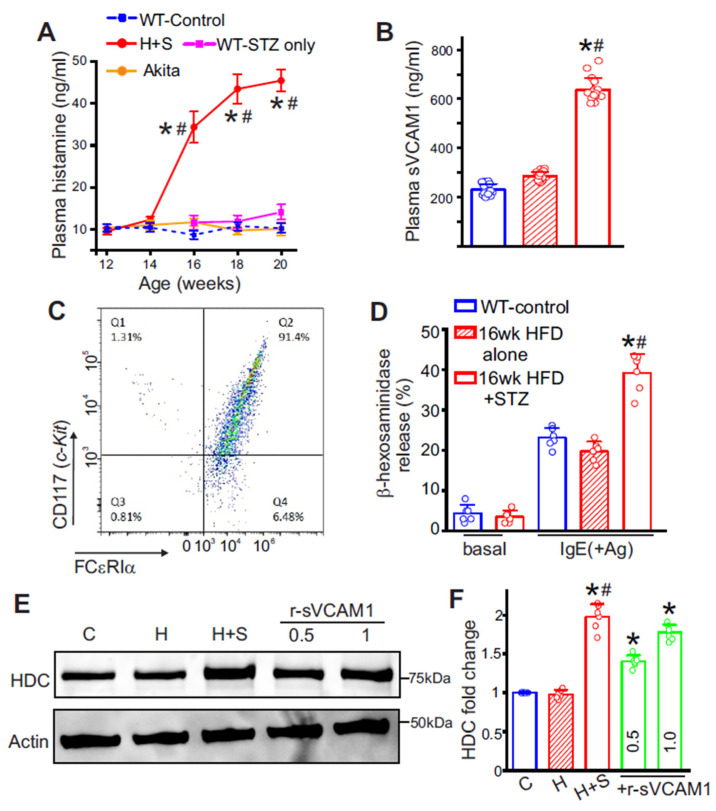
Increase in plasma histamine and sVCAM1 in diabetic mice is due to mast cell activation. (**A**) Plasma histamine levels in different groups of mice assessed from week 12 to week 20 of age. n = 8 for each time point, * *p* < 0.05 vs. control and # *p* < 0.05 vs. STZ only. (**B**) Plasma sVCAM1 levels at 16 weeks in H and H + S, n = 15 each, * *p* < 0.05 vs. respective control and # *p* < 0.05 vs. H only. (**C**) Representative flow cytometry analysis of peritoneal mast cell isolates showing a high percentage of cKit- and FCεRIα-positive cells. (**D**) Antigen-dependent degranulation assay in mast cells of different groups. n = 6 each. Basal unstimulated values are shown only for reference. * *p* < 0.05 vs. respective control and # *p* < 0.05 vs. H only. (**E**) Representative Western blot for HDC and Actin from MCs of different groups. Original western blots are shown in the Supplemental Figure S1. (**F**) Mean data. n= 6 for each, * *p* < 0.05 vs. respective control and # *p* < 0.05 vs. H only. Note: H—HFD only, H + S—HFD + STZ, C—Control, +r-sVCAM1—control cells treated with recombinant sVCAM1. Numerals above the blot and in the bar graph represent the concentration of r-sVCAM1 in µg/mL.

In HFD + STZ mice (T2D mice), plasma histamine rose sharply beginning at week 16, then reached a plateau over the remaining study period (Figure 2A). In contrast, Akita mice and WT mice receiving STZ without prior HFD showed no change in plasma histamine (Figure 2A). Histamine was also higher in *db*/*db* mice at 10 weeks of age (34.4 ± 2.43 ng/mL, n = 5), which similarly exhibited increased sVCAM1 (549.83 ± 56.23 ng/mL, n = 6). In age-matched comparisons, sVCAM1 was significantly higher in HFD plus STZ mice than in HFD alone (Figure 2B). Together, these data suggest that IR, rather than hyperglycemia or insulin deficiency alone, is associated with increased histamine levels and concurrent sVCAM1 elevation. Given the temporal overlap of these changes and the fact that MCs are the predominant source of circulating histamine, we next tested whether MCs contribute to this response.

### 3.3. Insulin Resistance Significantly Alters MC Degranulation Intensity

To test whether MC activation accounts for the rise in plasma histamine and to assess a role for sVCAM1, we first quantified MC degranulation. Peritoneal MCs were isolated and cultured as described in Methods. Flow cytometry showed robust c-Kit and FcεRIα expression, confirming MC identity and high purity (Figure 2C). With HFD alone, MC degranulation was lower than in controls (Figure 2D). However, post-IR, degranulation was ~2.0-fold higher (Figure 2D). This indicates that IR significantly alters MC homeostasis. We then investigated whether IR alone or sVCAM1 increased MC histamine synthesis.

### 3.4. sVCAM1 Increases Histidine Decarboxylase in MCs

Peritoneal MCs were analyzed by Western blot for histidine decarboxylase (HDC), the rate-limiting enzyme for histamine biosynthesis. HDC was significantly higher in MCs from WT T2D mice than from HFD alone controls (Figure 2E,F). To test causality, control MCs were exposed in vitro to recombinant sVCAM1 for 24 h at concentrations matching those observed in T2D plasma. Recombinant sVCAM1 increased HDC in a dose-dependent manner (Figure 2E,F). In parallel, r-sVCAM1-treated MCs showed no increase in beta hexosaminidase release compared with vehicle (Figure 2D). These results indicate that sVCAM1 preferentially upregulates the histamine biosynthesis in MCs, without directly enhancing degranulation pathways. Therefore, we next asked whether insulin resistance independently activates degranulation pathways in MCs.

### 3.5. Post-IR GSK3β Activation Drives MC Degranulation

**Figure 3:**
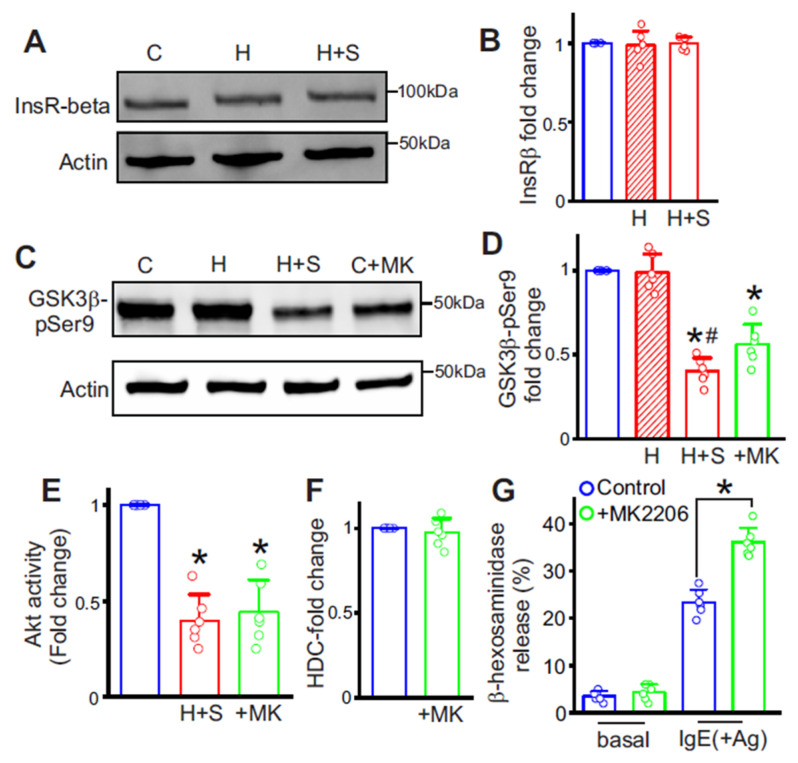
Insulin resistance triggers enhanced mast cell degranulation in diabetic mice. (**A**) Representative Western blot of Insulin receptor β subunit in MCs of different groups. (**B**) Mean data, n = 6 each. (**C**) Representative Western blot of GSK3β-pSer9 in MCs of different groups. Original western blots are shown in the Supplemental Figure S2. (**D**) Mean data, n = 6 each. * *p* < 0.05 vs. control and # *p* < 0.05 vs. HFD only. (**E**) Mean total Akt kinase activity in H + S and MK2206-treated control cells compared to control. * *p* < 0.05 vs. control. (**F**) Mean data of HDC protein expression after MK2206 treatment of control MCs. n = 6. (**G**) Antigen-dependent degranulation assay in control MCs treated with MK2206. Basal and stimulated control values are in blue, while MK2206 basal and treated values are in pink. * *p* < 0.05 vs. control. Note: MK2206 treatment was performed only in naïve cells from control WT mice. H—HFD only, H + S—HFD + STZ.

Insulin receptor signaling typically activates Akt, which phosphorylates and inhibits GSK3β at Ser9; this brake on GSK3β is impaired during IR across multiple tissues. We assessed MCs by Western blot for insulin receptor β subunit (InsRβ) and for pGSK3β-Ser9 (the inactive form of GSK3β). InsRβ expression was unchanged in HFD alone and in T2D (Figure 3A,B), whereas pGSK3β-Ser9 was significantly reduced after STZ, consistent with increased GSK3β activity in MCs (Figure 3C,D). Akt kinase activity was significantly lower in cells from HFD + STZ mice (Figure 3E), which supports the increased GSK3β activity. Pharmacologic Akt blockade with MK2206 in control MCs for 24 h likewise lowered Akt activity (Figure 3E), and pGSK3β-Ser9 (Figure 3C,D), and enhanced MC degranulation intensity (Figure 3G). In contrast, MK2206 did not alter HDC expression (Figure 3F). These findings indicate that IR-driven GSK3β activation augments MC degranulation but does not upregulate histamine synthesis.

### 3.6. Histamine Alters Histamine Receptor Expression in Cerebral Arteries and Disrupts Endothelial Barrier Integrity

**Figure 4:**
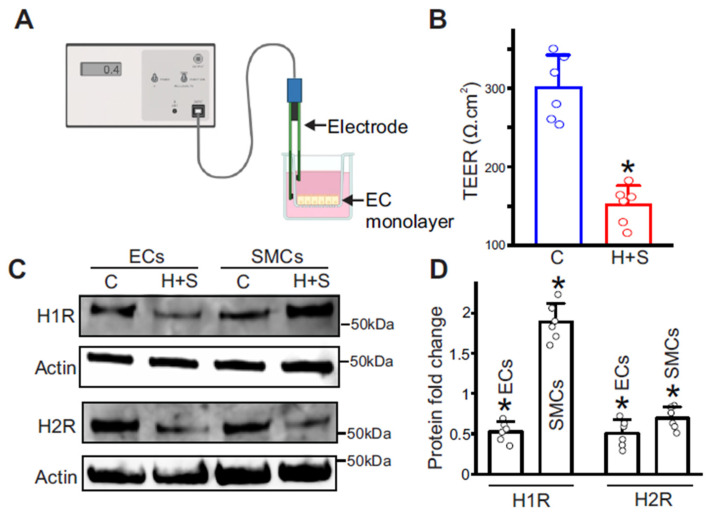
Histamine alters BBB integrity and histamine receptor expression in cerebral arteries. (**A**) Schematic of the TEER assay performed in isolated cerebral artery endothelial cells. (**B**) TEER measurements in control and 21-week-old HFD + STZ mice, n = 6 each. Each data point is the mean of three measurements. * *p* < 0.05 vs. the age-matched control. (**C**) Representative Western blots of H1 receptor, H2 receptor, and Actin in endothelial cells (ECs) and smooth muscle cells (SMCs) isolated from cerebral arteries of control and 21-week-old HFD + STZ mice. Original western blots are shown in the Supplemental Figure S3. (**D**) Mean data, n = 6 each, * *p* < 0.05 vs. the respective age-matched controls.

To test whether histamine contributes to cerebrovascular dysfunction in T2D, we quantified barrier integrity using an in vitro BBB model with primary cerebral artery endothelial monolayers. Transendothelial electrical resistance (TEER) was measured at 37 °C and corrected for insert area and blank resistance. Diabetic EC monolayers showed approximately a twofold reduction in TEER relative to controls (Figure 4A,B), indicating increased permeability and impaired BBB integrity. TEER values were stable across independent isolations, and the deficit persisted after media exchange, consistent with sustained barrier dysregulation. These findings support a model in which the T2D milieu, characterized by elevated histamine, weakens endothelial junctions and increases paracellular leak. Because histamine can cause vasoconstriction or vasodilation depending on receptor subtype and cell type, we examined receptor expression in T2D cerebral arteries. In vascular smooth muscle cells from 21-week-old T2D mice, H1R was markedly upregulated, and H2R was decreased. In endothelial cells, both H1R and H2R were downregulated (Figure 4C,D). This redistribution likely favors H1R-driven smooth muscle contraction (see Section 3.7) while diminishing endothelium-dependent, H2R-mediated relaxation.

### 3.7. Neutralization of sVCAM1 and Inhibition of GSK3β Stabilize Diabetic MCs and Improve Cerebrovascular Function

**Figure 5:**
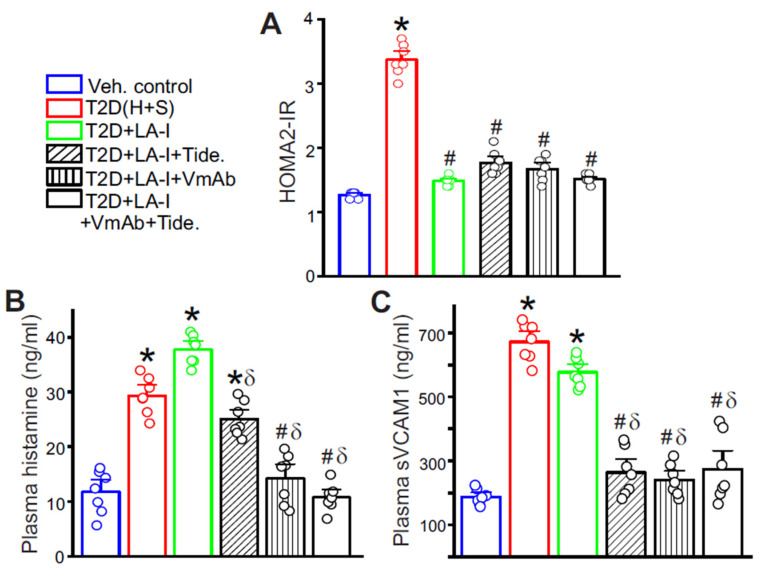
Glycemic control alone is insufficient to stabilize diabetic mast cells. Legends for the different treatment groups: Veh. Control-Vehicle control, LA-I-Long-acting Insulin, VmAb-VCAM1 monoclonal antibody, Tide.-Tideglusib. T2D refers to the H + S mice. (**A**) HOMA2-IR values calculated from glucose and insulin values of the respective treatment groups. n = 7 each, * *p* < 0.05 vs. Veh. control and # *p* < 0.05 vs. T2D (untreated). (**B**) Plasma histamine and (**C**) Plasma sVCAM1 levels in the different treatment groups. n = 7 each, * *p* < 0.05 vs. Veh. Control, # *p* < 0.05 vs. T2D (untreated), and δ *p* < 0.05 vs. T2D + LA-I alone.

Given the roles of sVCAM1 and GSK3β identified above, we questioned whether neutralizing sVCAM1 and inhibiting GSK3β would improve vascular outcomes in T2D. We treated mice with a monoclonal anti-VCAM1 antibody (VmAb) and the GSK3β antagonist Tideglusib, following the schedules detailed in Methods. Long-acting insulin (LA-I) alone and LA-I combined with either VmAb, Tideglusib, or both, lowered HOMA2-IR, confirming effective glycemic control (Figure 5A). However, LA-I alone did not lower circulating histamine or sVCAM1 (Figure 5B,C). In contrast, adding Tideglusib or VmAb produced partial reductions in plasma levels, while the combined regimen achieved the largest decrease in both mediators (Figure 5B,C). Together, these data indicate that glucose control alone is insufficient to reverse diabetic vascular dysfunction and that targeting the immune-vascular axis might provide additional benefit.

**Figure 6:**
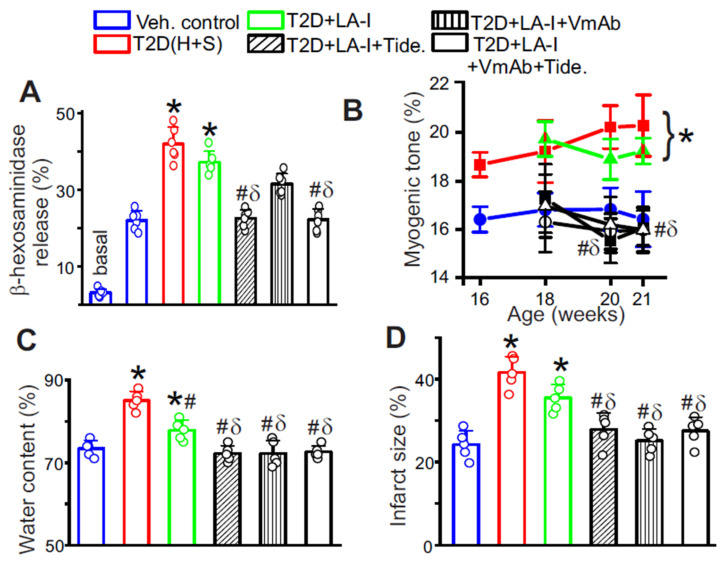
Stabilizing diabetic mast cells improves cerebrovascular function. (**A**) Antigen-dependent degranulation assay performed in MCs isolated from different treatment groups. The basal value of control is for reference only. n = 6 each. * *p* < 0.05 vs. Veh. Control, # *p* < 0.05 vs. T2D (untreated), and δ *p* < 0.05 vs. T2D + LA-I alone. (**B**) Myogenic tone of pressurized cerebral arteries at different time points during and after treatment. n = 5 for each time point. Legend for the treatment group includes: ● Control, ■ T2D, ▲T2D+LA-I, △ LA-I + Tideglusib, ○ LA-I + VmAb, ■ LA-I + Tideglusib + VmAb. * *p* < 0.05 vs. Veh. Control, # *p* < 0.05 vs. T2D (untreated), and δ *p* < 0.05 vs. T2D + LA-I alone. The flower bracket indicates that the time points for weeks 20 and 21 in T2D and T2D + LA-I are significantly different from those of the control. (**C**) Brain water content 24 h after MCAO and reperfusion, and (**D**) infarct size assessed by TTC staining of brain slices. n = 5 each, for B-D, * *p* < 0.05 vs. Veh. Control, # *p* < 0.05 vs. T2D (untreated), and δ *p* < 0.05 vs. T2D + LA-I alone.

Next, MCs from each treatment group were isolated and assessed for antigen-dependent degranulation by β-hexosaminidase release. LA-I alone produced only a modest reduction in degranulation (Figure 6A). Tideglusib, but not VmAb, suppressed β-hexosaminidase release, consistent with GSK3β primarily regulating MC releasability (Figure 6A). The combination regimen produced the greatest reduction in degranulation (Figure 6A), indicating that dual targeting limits both the degranulation program and the upstream inflammatory pathway that sustains MC activation in T2D. The effect of these treatments on the contractility of middle cerebral arteries was then measured using pressurized artery myography. Cerebral arteries from these mice were cannulated, and pressure-induced vasoconstriction was measured. Results indicated that in T2D mice, myogenic tone was elevated after IR onset compared with age-matched controls (Figure 6B), consistent with the increased smooth muscle H1R and reduced endothelial H2R observed by Western blot. LA-I alone did not alter the increased tone (Figure 6B). In contrast, either VmAb or Tideglusib, or both, significantly reduced cerebral artery tone and shifted the pressure response toward control values (Figure 6B). These findings indicate that targeting sVCAM1 and GSK3β corrects a histamine-biased constrictive state and improves cerebrovascular function. Finally, we evaluated stroke outcomes. In the transient dMCAO model, untreated T2D mice exhibited markedly greater brain edema and larger infarcts than controls (Figure 6C,D). LA-I alone produced only a modest reduction in edema and no meaningful change in infarct size (Figure 6C,D). In contrast, either VmAb or Tideglusib, or both, significantly reduced edema and infarct size (Figure 6C,D), indicating improved cerebrovascular resilience. These results suggest that adjunct targeting of sVCAM1 and GSK3β enhances vascular protection beyond glycemic control and may provide vasoprotection in settings of elevated stroke risk.

## 4. Discussion

In this study, we demonstrate that insulin resistance (IR) and elevated soluble VCAM-1 (sVCAM-1) converge on mast cells (MCs) to raise circulating histamine, linking metabolic dysfunction to immune activation and culminating in cerebrovascular injury. Beyond serving as a biomarker of endothelial activation, sVCAM-1 functions as a paracrine immunomodulator, while IR reshapes the circulating adhesion-molecule milieu. Together, these signals position MCs as key effectors at the immune-vascular interface in T2D. Here, we delineate these two complementary, yet distinct pathways that converge on MCs. First, IR diminishes Akt-dependent inhibitory phosphorylation of GSK3β at Ser9, thereby activating GSK3β in MCs and increasing degranulation intensity. Notably, this GSK3β signaling does not upregulate HDC and thus does not drive histamine synthesis. Second, building on our prior work showing that diabetic endothelial GSK3β activation increases VCAM-1 expression and ectodomain shedding [25], we tested endothelial-immune cross-talk and found that sVCAM1 directly increases HDC in native MCs. In summary, during T2D, IR promotes MC degranulation via GSK3β activation, while sVCAM1 promotes histamine production (HDC-dependent synthesis) to destabilize cerebrovascular function.

Current evidence suggests that MCs are involved in the pathogenesis of hypertension, atherosclerosis, obesity, and diabetes. MCs release pro-inflammatory mediators when activated [9,10,11] and are present within the cerebral architecture surrounding cerebral vessels in the meninges and close to the blood–brain barrier (BBB) [45,46,47]. The role of MCs in diabetes has been a subject of controversy, with evidence both supporting and contradicting their involvement in the development of obesity and IR [48,49,50,51,52]. As obesity and diabetes are now recognized as ‘chronic inflammatory diseases’ [6,7,8], there is a renewed focus on investigating the potential role of MCs in the development of vascular dysfunction in T2D. In this study, the causal role of MCs in T2D development was not evaluated; instead, we examined the effect of established IR on MC activation. Evidence for the proximal triggers of MC activation in T2D has been largely undefined. Similarly, although results across cohorts are not uniformly consistent, numerous reports associate higher circulating sICAM-1 and sVCAM-1 with prevalent CVD and adverse outcomes, with sVCAM-1 frequently emerging as a stronger predictor in select cardiovascular contexts [53,54,55,56,57,58,59] and in some analyses outperforming sICAM-1 [21,60]. In our study, circulating sVCAM-1 levels increased after the onset of IR and, when tested in vitro on native MCs, increased HDC expression.

ICAM-1 and VCAM-1 are membrane glycoproteins broadly expressed on endothelium and immune cells, where they coordinate leukocyte tethering, firm adhesion, and recruitment during inflammation, functions essential for an effective host response [53,61]. Under inflammatory stress, both proteins undergo ectodomain shedding, generating circulating soluble forms (sICAM-1 and sVCAM-1) detectable in plasma [19,20,21,22]. Beyond their canonical roles in cell–cell adhesion, these soluble adhesion molecules have been implicated in a broader range of immune activities, including the modulation of immune-cell activation states, thereby shaping the overall inflammatory tone [26,27,28]. Consistent with this, multiple studies place sICAM-1/sVCAM-1 within pathways governing endothelial-immune cross-talk [54,58,61,62]. The prominence of sVCAM-1 in CVD suggests opportunities to target either its generation or its interactions with leukocytes to slow plaque progression and reduce events.

In T2D, MCs release a broad repertoire of mediators, including serotonin (5-HT) [48], TNFα [63], proteases (tryptase [64,65] and chymase [66]), leukotrienes, and histamine, which can amplify vascular inflammation and increase vascular tone. Within this context, our data point to sVCAM1 as an upstream cue that biases MCs toward histamine biosynthesis rather than merely acute release. Specifically, sVCAM1 rose after IR onset. Our data show that when recombinant sVCAM-1 was applied to native MCs, HDC expression increased, consistent with a VLA-4 (α4β1)-dependent outside-in signaling program. Integrin engagement triggers FAK and Src, as well as the PI3K and MAPK cascades [23,24], which converge on transcriptional regulators such as NF-κB and CREB, capable of driving Hdc expression [67,68]. Our data refine the placement of GSK3β in MC biology by showing that GSK3β primarily tunes mediator release, whereas sVCAM1 drives histamine synthesis. GSK3β activation increased degranulation but did not increase HDC, supporting a distinct sVCAM1-to-VLA-4 biosynthetic pathway operating in parallel with IR-mediated GSK3β activation. The temporal sequence, with sVCAM1 rising after IR and followed by sustained elevation of plasma histamine, supports a causal role for endothelial-derived sVCAM1 in reprogramming the MC secretome. Consistent with this, histamine increased in IR-driven models such as *db*/*db* mice but not in T1D-Akita or STZ-alone WT mice, and sVCAM1 directly upregulated HDC in MCs. At the same time, IR-linked GSK3β activation enhanced degranulation, together implicating MCs as the predominant source of circulating histamine in this setting.

We also delineate how elevated histamine levels lead to cerebrovascular dysfunction. Histamine released by MCs is well-known for inducing vasodilation during allergic and anaphylactic reactions [14,15]. However, large-scale population studies involving patients with mastocytosis and severe asthma have uncovered that these patients have chronically elevated plasma histamine levels and, paradoxically, have a significantly increased risk of stroke [69,70,71,72]. Circulating histamine is increased in diabetes [16,17] and is postulated to contribute to the increased susceptibility of T2D patients to infectious and autoimmune diseases [16,18]. In our model, TEER assays showed an approximately twofold increase in permeability in diabetic endothelial monolayers, consistent with histamine-driven downregulation of junctional proteins and increased paracellular leak, which would be expected to promote leukocyte adhesion, transmigration, and endothelial stress [73,74,75]. Human studies show elevated plasma histamine in diabetes [16,76], and multiple rodent models indicate histamine dysregulation in diabetes [77,78]. Consistent with our findings, prior studies reported elevated plasma histamine in mice with HFD plus STZ-induced diabetes and in the genetic T2D strain, KK-Ay/TaJcl [79]. Further evidence suggests that, in aged mice after MCAO, intestinal MCs expanded and plasma histamine increased, with higher levels correlating with greater neuroinflammation and worse neurological outcomes [80]. Pharmacologic stabilization of gut MCs reduced circulating histamine, decreased neuroinflammation, and improved recovery [81]. At the level of vascular tone, histamine’s effect depends on the distribution of its receptors [82,83,84]. In week 21 T2D cerebral arteries, we observed H_1_R upregulated in vascular smooth muscle myocytes, with H_2_R not increased, while both H_1_R and H_2_R were downregulated in the endothelium. This redistribution favors smooth muscle constriction mediated by H_1_R and diminishes relaxation, which is usually supported by endothelial H_2_R signaling. Although our data define the receptor pattern and its association with increased myogenic tone, we did not test the upstream mechanisms that drive this remodeling. Both chronic histamine exposure and diabetes related metabolic stresses could contribute. Defining these pathways will require targeted experiments in future work.

Overall, these findings extend the view of sVCAM1 from a passive biomarker of endothelial stress to an active driver at the immune vascular interface. By directly engaging MCs to increase histamine, sVCAM1 provides a functional link between endothelial activation and immune effector output. In parallel, IR-dependent activation of GSK3β enhances MC degranulation. Together, these two independent pathways create a feed-forward circuit in which IR elevates sVCAM1 and activates GSK3β, leading to increased histamine synthesis and release, endothelial barrier weakening, and impaired cerebrovascular control. This was the rationale for using Tideglusib and VmAb together because they act at complementary, non-redundant nodes. Tideglusib suppresses the GSK3β pathway that heightens mast-cell degranulation, while VmAb blocks sVCAM1-VLA4 signaling that upregulates HDC and drives histamine synthesis.

VCAM-1 blockade has been previously shown to be disease-modifying in vivo. In ApoE^−/−^ mice, cross-reactive anti-VCAM-1 reduced plaque burden and inflammation [85]. In Ang II hypertension, VCAM-1 neutralization lowered blood pressure, diminished macrophage infiltration and oxidative stress, and improved vascular function [86]. Similar benefits were reported for hypertensive cardiac remodeling [87] and in allergic asthma models, where airway inflammation and remodeling were reduced [88].

In this study, glycemic control with long-acting insulin alone lowered HOMA2-IR but did not restore cerebral artery myogenic tone or improve stroke outcomes, indicating that improving metabolic indices is insufficient once diabetic vascular dysfunction is established. Adding either VmAb or Tideglusib to LA-I improved several readouts, consistent with each agent interrupting the same histamine-centered axis at a different step. VmAb primarily reduced the sVCAM1-linked histamine synthesis, whereas Tideglusib dampened the IR-linked GSK3β pathway that heightens MC releasability. In vivo, these actions likely intersect with additional effects that can narrow differences between single-agent and combination therapy. For example, Tideglusib can reduce endothelial sVCAM1 generation, as we reported previously, and VmAb may mitigate VCAM1-dependent endothelial activation and leukocyte recruitment in the post-stroke setting. Together, these overlapping vascular and inflammatory actions provide plausible reasons why LA-I plus either agent alone did not differ from the combination therapy across all functional outcomes.

Even so, the combined use of LA-I, Tideglusib, and VmAb is mechanistically favorable because it constrains both histamine production and histamine release, and therefore, can be expected to more completely suppress MC-driven vascular injury in T2D. Tideglusib targets degranulation by reducing MC releasability, while VmAb targets the biosynthetic arm by limiting sVCAM1-driven HDC upregulation and sustained histamine production. Acting together on these non-redundant nodes, the combination provides broader pathway coverage than either agent alone and is better positioned to deliver durable cerebrovascular protection.

Limitations and Future Directions: We initiated treatment after IR was established, which models therapeutic use rather than primary prevention. The study, therefore, cannot distinguish between preventive and reversal effects, and longer pre-IR intervention designs will be required to address the potential for disease modification. We also did not evaluate broader metabolic effects of our interventions, so off-target or beneficial metabolic actions of VmAb and Tideglusib remain undefined. Our stroke experiments were conducted over a relatively short timeline, whereas most human strokes arise after years of hypertension or diabetes. It is plausible that sustained VCAM1 blockade could yield an additive or even greater benefit in longer, more comorbid models. Finally, translation to humans will require careful consideration of histamine biology. Baseline plasma histamine in healthy humans is typically <1 ng/mL [89,90], whereas in rodents the normal range is broader and more variable, with lethality reported only above ~80–100 ng/mL [91,92,93,94]. These interspecies differences in baseline levels and tolerated ranges may alter dose–response relationships, receptor occupancy requirements, and the thresholds at which histamine affects vascular tone and barrier integrity. Future work will include ex vivo testing of human vascular tissue with patient-matched plasma, and prospective measurement of sVCAM1 and histamine in T2D cohorts to define clinically relevant exposure-response windows and to validate whether the mechanistic links identified here operate at human histamine concentrations.

## Data Availability

The original contributions presented in this study are included in the article/Supplementary Material. Further inquiries can be directed to the corresponding author.

## Supplementary Materials

The following supporting information can be downloaded at https://www.mdpi.com/article/10.3390/cells15050455/s1. Uncropped Western blots are shown in the Supplemental Figures S1–S3.

## Abbreviations

The following abbreviations are used in this manuscript:

T2D	Type 2 Diabetes
HFD	High-Fat Diet
STZ	Streptozotocin
IR	Insulin Resistance
MCs	Mast cells
ECs	Endothelial cells
LA-I	Long-acting Insulin

## References

[1] Centers for Disease Control and Prevention (2026). National Diabetes Statistics Report.

[2] Deshpande A.D., Harris-Hayes M., Schootman M. (2008). Epidemiology of diabetes and diabetes-related complications. Phys. Ther..

[3] Maric-Bilkan C. (2017). Sex differences in micro- and macro-vascular complications of diabetes mellitus. Clin. Sci..

[4] Kautzky-Willer A., Harreiter J. (2017). Sex and gender differences in therapy of type 2 diabetes. Diabetes Res. Clin. Pract..

[5] Chen R., Ovbiagele B., Feng W. (2016). Diabetes and Stroke: Epidemiology, Pathophysiology, Pharmaceuticals and Outcomes. Am. J. Med. Sci..

[6] Wu H., Ballantyne C.M. (2020). Metabolic Inflammation and Insulin Resistance in Obesity. Circ. Res..

[7] Rohm T.V., Meier D.T., Olefsky J.M., Donath M.Y. (2022). Inflammation in obesity, diabetes, and related disorders. Immunity.

[8] Donath M.Y., Shoelson S.E. (2011). Type 2 diabetes as an inflammatory disease. Nat. Rev. Immunol..

[9] Bot I., Shi G.-P., Kovanen P.T. (2015). Mast Cells as Effectors in Atherosclerosis. Arterioscler. Thromb. Vasc. Biol..

[10] Sun J., Sukhova G.K., Wolters P.J., Yang M., Kitamoto S., Libby P., MacFarlane L.A., Clair J.M.-S., Shi G.-P. (2007). Mast cells promote atherosclerosis by releasing proinflammatory cytokines. Nat. Med..

[11] Krystel-Whittemore M., Dileepan K.N., Wood J.G. (2015). Mast Cell: A Multi-Functional Master Cell. Front. Immunol..

[12] Bot I., Jager S.C.A.d., Zernecke A., Lindstedt K.A., Berkel T.J.C.v., Weber C., Biessen E.A.L. (2007). Perivascular Mast Cells Promote Atherogenesis and Induce Plaque Destabilization in Apolipoprotein E–Deficient Mice. Circulation.

[13] Kovanen P.T. (2019). Mast Cells as Potential Accelerators of Human Atherosclerosis-From Early to Late Lesions. Int. J. Mol. Sci..

[14] Kunder C.A., St John A.L., Abraham S.N. (2011). Mast cell modulation of the vascular and lymphatic endothelium. Blood.

[15] Theoharides T.C., Alysandratos K.D., Angelidou A., Delivanis D.A., Sismanopoulos N., Zhang B., Asadi S., Vasiadi M., Weng Z., Miniati A. (2012). Mast cells and inflammation. Biochim. Biophys. Acta.

[16] Pini A., Obara I., Battell E., Chazot P.L., Rosa A.C. (2016). Histamine in diabetes: Is it time to reconsider?. Pharmacol. Res..

[17] Shi M., Shi G.-P. (2012). Different Roles of Mast Cells in Obesity and Diabetes: Lessons from Experimental Animals and Humans. Front. Immunol..

[18] Kempuraj D., Caraffa A., Ronconi G., Lessiani G., Conti P. (2016). Are mast cells important in diabetes?. Pol. J. Pathol..

[19] Garton K.J., Gough P.J., Philalay J., Wille P.T., Blobel C.P., Whitehead R.H., Dempsey P.J., Raines E.W. (2003). Stimulated shedding of vascular cell adhesion molecule 1 (VCAM-1) is mediated by tumor necrosis factor-alpha-converting enzyme (ADAM 17). J. Biol. Chem..

[20] Bruno C.M., Valenti M., Bertino G., Ardiri A., Bruno F., Cunsolo M., Pulvirenti D., Neri S. (2008). Plasma ICAM-1 and VCAM-1 levels in type 2 diabetic patients with and without microalbuminuria. Minerva Med..

[21] Troncoso M.F., Ortiz-Quintero J., Garrido-Moreno V., Sanhueza-Olivares F., Guerrero-Moncayo A., Chiong M., Castro P.F., García L., Gabrielli L., Corbalán R. (2021). VCAM-1 as a predictor biomarker in cardiovascular disease. Biochim. Et Biophys. Acta (BBA)-Mol. Basis Dis..

[22] Schmidt A.M., Crandall J., Hori O., Cao R., Lakatta E. (1996). Elevated plasma levels of vascular cell adhesion molecule-1 (VCAM-1) in diabetic patients with microalbuminuria: A marker of vascular dysfunction and progressive vascular disease. Br. J. Haematol..

[23] Katoh K. (2025). Integrin and Its Associated Proteins as a Mediator for Mechano-Signal Transduction. Biomolecules.

[24] Pang X., He X., Qiu Z., Zhang H., Xie R., Liu Z., Gu Y., Zhao N., Xiang Q., Cui Y. (2023). Targeting integrin pathways: Mechanisms and advances in therapy. Signal Transduct. Target. Ther..

[25] Brishti M.A., Raghavan S., Lamar K., Singh U.P., Collier D.M., Leo M.D. (2023). Diabetic Endothelial Cell Glycogen Synthase Kinase 3β Activation Induces VCAM1 Ectodomain Shedding. Int. J. Mol. Sci..

[26] Boucher J., Kleinridders A., Kahn C.R. (2014). Insulin receptor signaling in normal and insulin-resistant states. Cold Spring Harb. Perspect. Biol..

[27] De Meyts P., De Groot L.J., Chrousos G., Dungan K., Feingold K.R., Grossman A., Hershman J.M., Koch C., Korbonits M., McLachlan R., New M. (2000). The Insulin Receptor and Its Signal Transduction Network. Endotext.

[28] Bandyopadhyay G., Standaert M.L., Galloway L., Moscat J., Farese R.V. (1997). Evidence for involvement of protein kinase C (PKC)-zeta and noninvolvement of diacylglycerol-sensitive PKCs in insulin-stimulated glucose transport in L6 myotubes. Endocrinology.

[29] Standaert M.L., Galloway L., Karnam P., Bandyopadhyay G., Moscat J., Farese R.V. (1997). Protein kinase C-zeta as a downstream effector of phosphatidylinositol 3-kinase during insulin stimulation in rat adipocytes. Potential role in glucose transport. J. Biol. Chem..

[30] Hamamdzic D., Fenning R.S., Patel D., Mohler E.R., Orlova K.A., Wright A.C., Llano R., Keane M.G., Shannon R.P., Birnbaum M.J. (2010). Akt pathway is hypoactivated by synergistic actions of diabetes mellitus and hypercholesterolemia resulting in advanced coronary artery disease. Am. J. Physiol. Heart Circ. Physiol..

[31] Geraldes P., King G.L. (2010). Activation of protein kinase C isoforms and its impact on diabetic complications. Circ. Res..

[32] Okon E.B., Chung A.W., Rauniyar P., Padilla E., Tejerina T., McManus B.M., Luo H., van Breemen C. (2005). Compromised arterial function in human type 2 diabetic patients. Diabetes.

[33] Raghavan S., Brishti M.A., Bernardelli A., Mata-Daboin A., Jaggar J.H., Leo M.D. (2024). Extracellular glucose and dysfunctional insulin receptor signaling independently upregulate arterial smooth muscle TMEM16A expression. Am. J. Physiol. Cell Physiol..

[34] Tolosa E., Litvan I., Hoglinger G.U., Burn D., Lees A., Andres M.V., Gomez-Carrillo B., Leon T., Del Ser T. (2014). A phase 2 trial of the GSK-3 inhibitor tideglusib in progressive supranuclear palsy. Mov. Disord. Off. J. Mov. Disord. Soc..

[35] Brishti M.A., Vazhappully Francis F., Leo M.D. (2025). Plasma Metabolomic Profiling Reveals Systemic Alterations in a Mouse Model of Type 2 Diabetes. Metabolites.

[36] Nagel J.M., Staffa J., Renner-Müller I., Horst D., Vogeser M., Langkamp M., Hoeflich A., Göke B., Kolligs F.T., Mantzoros C.S. (2010). Insulin glargine and NPH insulin increase to a similar degree epithelial cell proliferation and aberrant crypt foci formation in colons of diabetic mice. Horm. Cancer.

[37] Li Y., Zheng J., Shen Y., Li W., Liu M., Wang J., Zhu S., Wu M. (2018). Comparative Study of Liraglutide and Insulin Glargine on Glycemic Control and Pancreatic β-Cell Function in db/db Mice. Med. Sci. Monit..

[38] Tsvilovskyy V., Solis-Lopez A., Öhlenschläger K., Freichel M. (2018). Isolation of Peritoneum-derived Mast Cells and Their Functional Characterization with Ca^2+^-imaging and Degranulation Assays. JoVE.

[39] Meurer S.K., Neß M., Weiskirchen S., Kim P., Tag C.G., Kauffmann M., Huber M., Weiskirchen R. (2016). Isolation of Mature (Peritoneum-Derived) Mast Cells and Immature (Bone Marrow-Derived) Mast Cell Precursors from Mice. PLoS ONE.

[40] Nasoohi S., Tayefeh Ghahremani P., Alehossein P., Elyasizadeh S., BaniArdalan S., Ismael S., Vatanpour H., Ahmadiani A., Ishrat T. (2023). The p75 neurotrophin receptor inhibitor, LM11A-31, ameliorates acute stroke injury and modulates astrocytic proNGF. Exp. Neurol..

[41] Alhusban A., Kozak A., Pillai B., Ahmed H., Sayed M.A., Johnson M.H., Ishrat T., Ergul A., Fagan S.C. (2017). Mechanisms of acute neurovascular protection with AT1 blockade after stroke: Effect of prestroke hypertension. PLoS ONE.

[42] Furman B.L. (2021). Streptozotocin-Induced Diabetic Models in Mice and Rats. Curr. Protoc..

[43] Gheibi S., Kashfi K., Ghasemi A. (2017). A practical guide for induction of type-2 diabetes in rat: Incorporating a high-fat diet and streptozotocin. Biomed. Pharmacother..

[44] Nagy C., Einwallner E. (2018). Study of In Vivo Glucose Metabolism in High-fat Diet-fed Mice Using Oral Glucose Tolerance Test (OGTT) and Insulin Tolerance Test (ITT). J. Vis. Exp..

[45] McKittrick C.M., Lawrence C.E., Carswell H.V.O. (2015). Mast Cells Promote Blood Brain Barrier Breakdown and Neutrophil Infiltration in a Mouse Model of Focal Cerebral Ischemia. J. Cereb. Blood Flow. Metab..

[46] Arac A., Grimbaldeston M.A., Galli S.J., Bliss T.M., Steinberg G.K. (2019). Meningeal Mast Cells as Key Effectors of Stroke Pathology. Front. Cell Neurosci..

[47] Silver R., Silverman A.J., Vitković L., Lederhendler II. (1996). Mast cells in the brain: Evidence and functional significance. Trends Neurosci..

[48] Yabut J.M., Desjardins E.M., Chan E.J., Day E.A., Leroux J.M., Wang B., Crane E.D., Wong W., Morrison K.M., Crane J.D. (2020). Genetic deletion of mast cell serotonin synthesis prevents the development of obesity and insulin resistance. Nat. Commun..

[49] Gutierrez D.A., Muralidhar S., Feyerabend T.B., Herzig S., Rodewald H.-R. (2015). Hematopoietic Kit Deficiency, rather than Lack of Mast Cells, Protects Mice from Obesity and Insulin Resistance. Cell Metab..

[50] Zhang J., Shi G.P. (2012). Mast cells and metabolic syndrome. Biochim. Et Biophys. Acta.

[51] Liu J., Divoux A., Sun J., Zhang J., Clément K., Glickman J.N., Sukhova G.K., Wolters P.J., Du J., Gorgun C.Z. (2009). Genetic deficiency and pharmacological stabilization of mast cells reduce diet-induced obesity and diabetes in mice. Nat. Med..

[52] Gurung P., Moussa K., Adams-Huet B., Devaraj S., Jialal I. (2019). Increased mast cell abundance in adipose tissue of metabolic syndrome: Relevance to the proinflammatory state and increased adipose tissue fibrosis. Am. J. Physiol.-Endocrinol. Metab..

[53] Kallmann B.A., Hummel V., Toyka K.V., Rieckmann P., Hommes O.R., Comi G. (2004). Soluble VCAM-1 Release Indicates Inflammatory Blood-Brain Barrier Pathology and Further Modulates Adhesion. Early Indicators Early Treatments Neuroprotection in Multiple Sclerosis.

[54] Kotteas E.A., Boulas P., Gkiozos I., Tsagkouli S., Tsoukalas G., Syrigos K.N. (2014). The Intercellular Cell Adhesion Molecule-1 (ICAM-1) in Lung Cancer: Implications for Disease Progression and Prognosis. Anticancer Res..

[55] Champagne B., Tremblay P., Cantin A., St. Pierre Y. (1998). Proteolytic Cleavage of ICAM-1 by Human Neutrophil Elastase. J. Immunol..

[56] Witte D.R., Broekmans W.M., Kardinaal A.F., Klöpping-Ketelaars I.A., van Poppel G., Bots M.L., Kluft C., Princen J.M. (2003). Soluble intercellular adhesion molecule 1 and flow-mediated dilatation are related to the estimated risk of coronary heart disease independently from each other. Atherosclerosis.

[57] Semaan H.B., Gurbel P.A., Anderson J.L., Muhlestein J.B., Carlquist J.F., Horne B.D., Serebruany V.L. (2000). Soluble VCAM-1 and E-Selectin, but Not ICAM-1 Discriminate Endothelial Injury in Patients with Documented Coronary Artery Disease. Cardiology.

[58] Demerath E., Towne B., Blangero J., Siervogel R.M. (2001). The relationship of soluble ICAM-1, VCAM-1, P-selectin and E-selectin to cardiovascular disease risk factors in healthy men and women. Ann. Hum. Biol..

[59] Hwang S.J., Ballantyne C.M., Sharrett A.R., Smith L.C., Davis C.E., Gotto A.M., Boerwinkle E. (1997). Circulating adhesion molecules VCAM-1, ICAM-1, and E-selectin in carotid atherosclerosis and incident coronary heart disease cases: The Atherosclerosis Risk In Communities (ARIC) study. Circulation.

[60] Hegazy G.A., Awan Z., Hashem E., Al-Ama N., Abunaji A.B. (2020). Levels of soluble cell adhesion molecules in type 2 diabetes mellitus patients with macrovascular complications. J. Int. Med. Res..

[61] Ramos T.N., Bullard D.C., Barnum S.R. (2014). ICAM-1: Isoforms and Phenotypes. J. Immunol..

[62] Bui T.M., Wiesolek H.L., Sumagin R. (2020). ICAM-1: A master regulator of cellular responses in inflammation, injury resolution, and tumorigenesis. J. Leukoc. Biol..

[63] Yao X., Wang X., Zhang R., Kong L., Fan C., Qian Y. (2025). Dysregulated mast cell activation induced by diabetic milieu exacerbates the progression of diabetic peripheral neuropathy in mice. Nat. Commun..

[64] Deliargyris E.N., Upadhya B., Sane D.C., Dehmer G.J., Pye J., Smith S.C., Boucher W.S., Theoharides T.C. (2005). Mast cell tryptase: A new biomarker in patients with stable coronary artery disease. Atherosclerosis.

[65] Zhi X., Xu C., Zhang H., Tian D., Li X., Ning Y., Yin L. (2013). Tryptase promotes atherosclerotic plaque haemorrhage in ApoE-/- mice. PLoS ONE.

[66] Bot I., Bot M., van Heiningen S.H., van Santbrink P.J., Lankhuizen I.M., Hartman P., Gruener S., Hilpert H., van Berkel T.J.C., Fingerle J. (2010). Mast cell chymase inhibition reduces atherosclerotic plaque progression and improves plaque stability in ApoE^−/−^ mice. Cardiovasc. Res..

[67] Huang H., Li Y., Liang J., Finkelman F.D. (2018). Molecular Regulation of Histamine Synthesis. Front. Immunol..

[68] Zhang Z., Höcker M., Koh T.J., Wang T.C. (1996). The human histidine decarboxylase promoter is regulated by gastrin and phorbol 12-myristate 13-acetate through a downstream cis-acting element. J. Biol. Chem..

[69] Broesby-Olsen S., Farkas D.K., Vestergaard H., Hermann A.P., Møller M.B., Mortz C.G., Kristensen T.K., Bindslev-Jensen C., Sørensen H.T., Frederiksen H. (2016). Risk of solid cancer, cardiovascular disease, anaphylaxis, osteoporosis and fractures in patients with systemic mastocytosis: A nationwide population-based study. Am. J. Hematol..

[70] Indhirajanti S., van Daele P.L.A., Bos S., Mulder M.T., Bot I., Roeters van Lennep J.E. (2018). Systemic mastocytosis associates with cardiovascular events despite lower plasma lipid levels. Atherosclerosis.

[71] Iribarren C., Tolstykh I.V., Miller M.K., Sobel E., Eisner M.D. (2012). Adult asthma and risk of coronary heart disease, cerebrovascular disease, and heart failure: A prospective study of 2 matched cohorts. Am. J. Epidemiol..

[72] Hermans M., Lennep J.R.V., van Daele P., Bot I. (2019). Mast Cells in Cardiovascular Disease: From Bench to Bedside. Int. J. Mol. Sci..

[73] Ashina K., Tsubosaka Y., Nakamura T., Omori K., Kobayashi K., Hori M., Ozaki H., Murata T. (2015). Histamine Induces Vascular Hyperpermeability by Increasing Blood Flow and Endothelial Barrier Disruption In Vivo. PLoS ONE.

[74] Bañuelos-Cabrera I., Valle-Dorado M.G., Aldana B.I., Orozco-Suárez S.A., Rocha L. (2014). Role of histaminergic system in blood-brain barrier dysfunction associated with neurological disorders. Arch. Med. Res..

[75] Yue J., Tan Y., Huan R., Guo J., Yang S., Deng M., Xiong Y., Han G., Liu L., Liu J. (2023). Mast cell activation mediates blood-brain barrier impairment and cognitive dysfunction in septic mice in a histamine-dependent pathway. Front. Immunol..

[76] Gill D.S., Barradas M.A., Fonseca V.A., Dandona P. (1989). Plasma histamine concentrations are elevated in patients with diabetes mellitus and peripheral vascular disease. Metabolism.

[77] Lee B.J., Byeon H.E., Cho C.S., Kim Y.H., Kim J.H., Che J.H., Seok S.H., Kwon J.W., Kim J.H., Lee K. (2020). Histamine causes an imbalance between pro-angiogenic and anti-angiogenic factors in the retinal pigment epithelium of diabetic retina via H4 receptor/p38 MAPK axis. BMJ Open Diabetes Res. Care.

[78] Gill D.S., Thompson C.S., Dandona P. (1990). Histamine synthesis and catabolism in various tissues in diabetic rats. Metabolism.

[79] Horikawa T., Hiramoto K., Goto K., Sekijima H., Ooi K. (2021). Differences in the mechanism of type 1 and type 2 diabetes-induced skin dryness by using model mice. Int. J. Med. Sci..

[80] Blasco M.P., Chauhan A., Honarpisheh P., Ahnstedt H., d’Aigle J., Ganesan A., Ayyaswamy S., Blixt F., Venable S., Major A. (2020). Age-dependent involvement of gut mast cells and histamine in post-stroke inflammation. J. Neuroinflamm..

[81] Conesa M.P.B., Blixt F.W., Peesh P., Khan R., Korf J., Lee J., Jagadeesan G., Andersohn A., Das T.K., Tan C. (2023). Stabilizing histamine release in gut mast cells mitigates peripheral and central inflammation after stroke. J. Neuroinflamm..

[82] Komarova Y.A., Kruse K., Mehta D., Malik A.B. (2017). Protein Interactions at Endothelial Junctions and Signaling Mechanisms Regulating Endothelial Permeability. Circ. Res..

[83] Zdravkovic V., Pantovic S., Rosic G., Tomic-Lucic A., Zdravkovic N., Colic M., Obradovic Z., Rosic M. (2011). Histamine Blood Concentration in Ischemic Heart Disease Patients. J. Biomed. Biotechnol..

[84] Jin H., Koyama T., Hatanaka Y., Akiyama S., Takayama F., Kawasaki H. (2006). Histamine-induced vasodilation and vasoconstriction in the mesenteric resistance artery of the rat. Eur. J. Pharmacol..

[85] Park J.G., Ryu S.Y., Jung I.H., Lee Y.H., Kang K.J., Lee M.R., Lee M.N., Sonn S.K., Lee J.H., Lee H. (2013). Evaluation of VCAM-1 antibodies as therapeutic agent for atherosclerosis in apolipoprotein E-deficient mice. Atherosclerosis.

[86] Yin L., Bai J., Yu W.J., Liu Y., Li H.H., Lin Q.Y. (2022). Blocking VCAM-1 Prevents Angiotensin II-Induced Hypertension and Vascular Remodeling in Mice. Front. Pharmacol..

[87] Qiu Z.Y., Yu W.J., Bai J., Lin Q.Y. (2022). Blocking VCAM-1 ameliorates hypertensive cardiac remodeling by impeding macrophage infiltration. Front. Pharmacol..

[88] Lee J.H., Sohn J.H., Ryu S.Y., Hong C.S., Moon K.D., Park J.W. (2013). A novel human anti-VCAM-1 monoclonal antibody ameliorates airway inflammation and remodelling. J. Cell Mol. Med..

[89] Friedman B.S., Steinberg S.C., Meggs W.J., Kaliner M.A., Frieri M., Metcalfe D.D. (1989). Analysis of plasma histamine levels in patients with mast cell disorders. Am. J. Med..

[90] Maintz L., Novak N. (2007). Histamine and histamine intolerance. Am. J. Clin. Nutr..

[91] Noguchi K., Ishida J., Kim J.-D., Muromachi N., Kako K., Mizukami H., Lu W., Ishimaru T., Kawasaki S., Kaneko S. (2020). Histamine receptor agonist alleviates severe cardiorenal damages by eliciting anti-inflammatory programming. Proc. Natl. Acad. Sci. USA.

[92] Musio S., Gallo B., Scabeni S., Lapilla M., Poliani P.L., Matarese G., Ohtsu H., Galli S.J., Mantegazza R., Steinman L. (2006). A Key Regulatory Role for Histamine in Experimental Autoimmune Encephalomyelitis: Disease Exacerbation in Histidine Decarboxylase-Deficient Mice. J. Immunol..

[93] Naganuma F., Nakamura T., Yoshikawa T., Iida T., Miura Y., Kárpáti A., Matsuzawa T., Yanai A., Mogi A., Mochizuki T. (2017). Histamine N-methyltransferase regulates aggression and the sleep-wake cycle. Sci. Rep..

[94] Oguri S., Motegi K., Endo Y. (2003). Augmented lipopolysaccharide-induction of the histamine-forming enzyme in streptozotocin-induced diabetic mice. Biochim. Et Biophys. Acta (BBA)-Mol. Basis Dis..

