# Supplementary material for: Targeting Soluble VCAM1 and GSK3β Improves Cerebrovascular Function and Reduces Stroke Pathology in Diabetic Mice
Source: Cells. 2026 Mar 4;15(5):455. doi: 10.3390/cells15050455 (PMC12984772; doi:10.3390/cells15050455)

Figure S1

Fig 2E

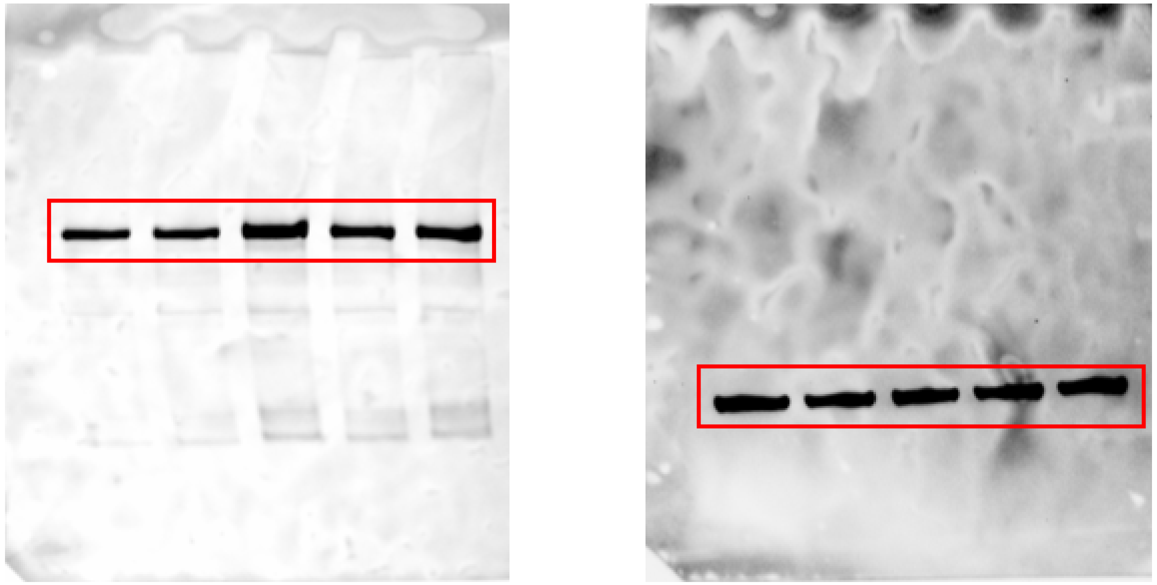

Figure S2

Fig 3A

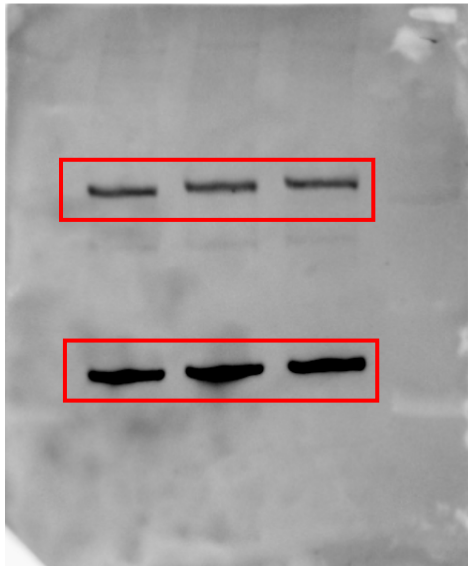

Note: Blot was dual-probed with Insulin receptor antibody (followed by anti-rabbit secondary) and Actin antibody (followed by anti-mouse secondary)

Fig 3C

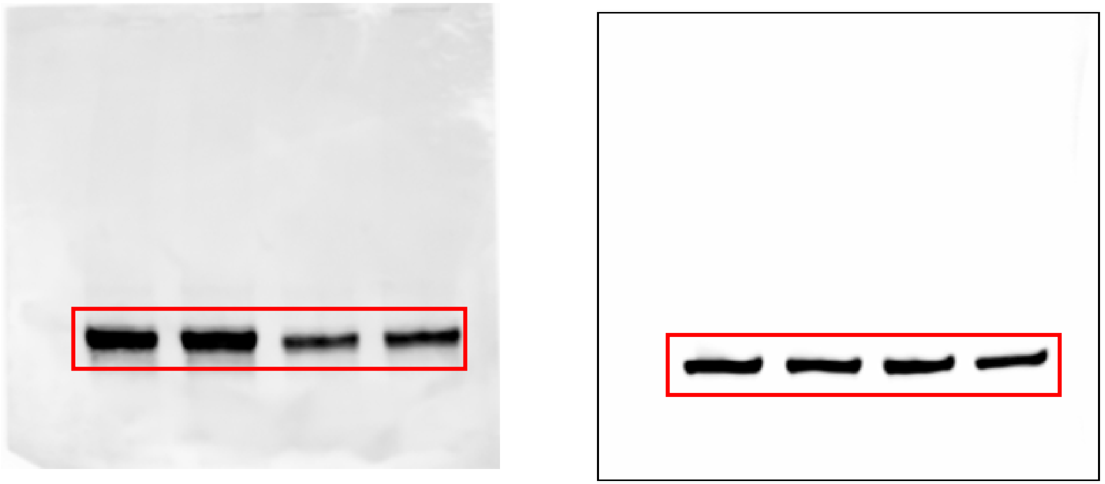

Figure S3

Fig 4C

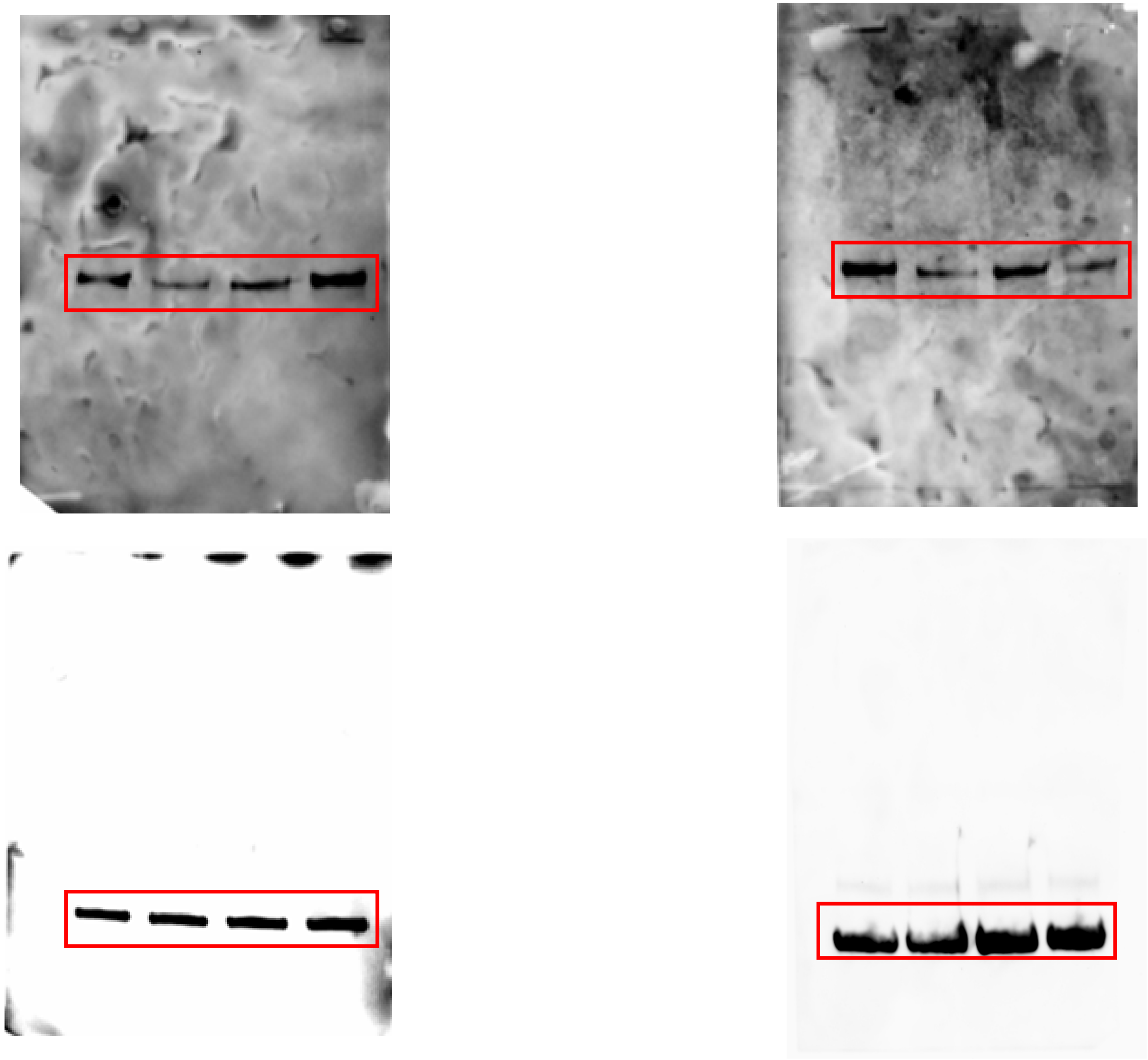

Supplement: Supplementary file 1 [file cells-15-00455-s001.zip › cells-4076981-supplementary.pdf]
